# Cost effectiveness of virtual reality game compared to clinic based McKenzie extension therapy for chronic non-specific low back pain

**DOI:** 10.1177/20494637221109108

**Published:** 2022-06-16

**Authors:** Francis Fatoye, Tadesse Gebrye, Chidozie Emmanuel Mbada, Clara T Fatoye, Moses O Makinde, Salami Ayomide, Blessing Ige

**Affiliations:** 1 Department of Health Professions, 5289 Manchester Metropolitan University, Manchester, UK; 2 Department of Medical Rehabilitation, College of Health Sciences, 54715 Obafemi Awolowo University, Ile-Ife, Nigeria

**Keywords:** Cost-effectiveness analysis, low back pain, McKenzie extension, virtual reality, video games

## Abstract

**Background:**

Low-back pain (LBP) is a major public health problem globally and its direct and indirect healthcare costs are growing rapidly. Virtual reality involving the use of video games or non-game applications are alternatives to conventional face-to-face physical therapy for LBP. The purpose of this study was to assess the cost-effectiveness of Back Extension-Virtual Reality Game (BE-VRG) compared to Clinic-based McKenzie therapy (CBMT) for chronic non-specific LBP in Nigeria.

**Methods:**

Patients with chronic non-specific LBP were randomised into either BE-VRG or CBMT group. Patients’ level of disability was assessed using Oswestry Disability Index (ODI) at week 4 and week 8. ODI was mapped to SF-6D to generate quality adjusted life years (QALYs) used for cost-effectiveness analysis. Resource use and costs were assessed based on rehabilitation services from a healthcare perspective. Cost-effectiveness analysis which included direct healthcare costs was conducted. Incremental cost per QALY was also calculated.

**Results:**

Forty-six patients (BE-VRG, *n* = 22; CBMT, *n* = 24) with the mean (±SD) age of 32.6 ± (11.5) years for BE-VRG and 48.8 ± (10.2) years for CBMT intervention completed in this study. The mean direct health costs per patient were USD100.67 and USD106.3 for BE-VRG and CBMT, respectively. The mean quality adjusted life years at week 4 and week 8 were (BE-VRG, 0.0574 ± (0.002); CBMT, 0.0548 ± (0.002)); and (BE-VRG; 0.116 ± (0.002); CBMT; 0.114 ± (0.004)), respectively. Incremental cost-effectiveness ratio showed that BE-VRG arm was less costly and more effective than CBMT.

**Conclusion:**

The findings of this study suggest that BE-VRG was cost saving for chronic non-specific LBP compared to CBMT. This evidence could guide policy makers, payers and clinicians in evaluating BE-VRG as a treatment option for people with chronic non-specific LBP.

## Introduction

Chronic low-back pain (LBP) is pain in the lower back persisting for at least 12 weeks.^1^ LBP is a major public health problem globally and typically classified as being specific or nonspecific.^2^ Nonspecific LBP refers to pain with unknown origin.^3^ In 2017, LBP was responsible for 577 million affected people and 64.9 million years lived with worldwide.^4^ In 2019, the personal and societal impact of LBP in the Netherlands were €4875 and €4315 per patient, respectively.^5^ Parallel to this, in 2004 indirect cost of US$ 7,400,000,000 due to LBP was spent at national level in United States of America.^6^ Musculoskeletal problems with complaints of back pain were also one in seven of all the recorded consultations in the United Kingdom in 2006.^7^ Therefore, LBP is not only a health problem but also a socioeconomic problem.

A range of management strategies are recommended for LBP including surgery, pharmacological and non-pharmacological therapies.^8^ Non-surgical and non-pharmacological therapies such as physiotherapy interventions are the most widely used type of management strategies for LBP.^9^ Indeed, exercise therapy is arguably the cheapest physiotherapeutic intervention and one in which the patient has some measure of direct control.^10^ However, it remains inconclusive which type of exercise is better than the other,^9^ leading to a proliferation of exercise programmes with limited evidence on their effectiveness and cost-effectiveness. In some Western nations, one of the most frequently used and effective type of physiotherapy for patients with long-term LBP is the McKenzie exercise.^11^ A recent systematic review on the effectiveness of the McKenzie method in the management of patients with chronic low back pain reported that the McKenzie method is a successful treatment to reduce pain in the short term and in enhancing function in the long term.^12^ The implementation of McKenzie exercise may have some limitation in remote areas due to the lack of specialized training of patients to help themselves in their own time.^13,14^ Further, there is a scarcity of health services in the rural areas of Africa.^15,16^ The problem of distance and time to receive healthcare can be solved by using telerehabilitation.^17^ Telerehabilitation, a mobile phone based application, used to treat LBP patients. In addition to mobile phones patients with LBP could also use a virtual reality game (VRG). It involves the use of video games or non-game applications.^18^

Evidence suggests that Back Extension-Virtual Reality Game (BE-VRG) is comparable with the McKenzie extension therapy in its effect on pain and pain-related characteristics, disability and quality of life.^16^ The failure to investigate the cost-effectiveness of BE-VRG may limit its potential in the management of LBP. Therefore, this study examined the cost-effectiveness of BE-VRG compared to a conventional face-to-face intervention for chronic non-specific LBP.

## Material and methods

### Design overview

This study utilised a randomised controlled trial design. Patients were randomised into the experimental group received the BE-VRG. Whereas, the control group received the Clinic-Based Mckenzie Therapy (CBMT). In order to ensure equal-sized treatment groups, random permuted blocks was used,^19^ a block size of four was chosen for this study. Computer-generated block permutations for four blocks of two group (i.e. 4 factorial - (4!)/((2!)(2!)) yielded 24 permutation (such as AABB, ABAB and all other possible restricted permutations).^19^ The process of drawing block permuted sequence and randomisation was repeated as participants were recruited. Single blind approach where only the researchers know whether a patient was receiving conventional treatment or the new one, in order to reduce bias.

Ethical approval for the study was obtained from the Health Research Ethics Committee of the Institute of Public Health, Obafemi Awolowo University, Ile-Ife, Nigeria (IPHOAU/12/885). Informed consent was obtained from all participants following full disclosure of the objective of the study.

### Participants

Participants with chronic non-specific LBP were recruited from selected physiotherapy facilities, namely: Obafemi Awolowo University (OAU) Teaching Hospital, Ile Ife, Nigeria (OAUTHC); Department of Medical Rehabilitation, OAU and Ladoke Akintola University of Technology Teaching Hospital, Osogbo, Nigeria. The sample size for the study was based on a statistical calculation comparing two means – sample size.^20^ The calculator uses the *n* = (Zα/2+Zβ)2 *2*σ2/d2, where Zα/2 is the critical value of the Normal distribution at α/2 (e.g. for a confidence level of 95%, α is 0.05 and the critical value is 1.96), Zβ is the critical value of the Normal distribution at β (e.g. for a power of 80%, β is 0.2 and the critical value is 0.84), σ2 is the population variance and d is the difference to be detected. Therefore, using a confidence level of 95%, power of 80%, population variance of 100 and a difference of 5, therefore *n* = 16 per group. Accounting for 20% possible attrition, approximately 20 participants per group will be required (*N* = 40). A total of 72 patients were assessed for eligibility in this study. 57 of them were found eligible and then randomized into both groups. However, only 46 of the eligible participants completed the study. Treatment discontinuation in both groups were mainly due to lack of interest, especially due to noticeable improvement in patients’ conditions.

Eligible participants for this study were patients with LBP of not less than 3 months. The standard McKenzie Institute Lumbar Spine Assessment Algorithm was used to determine eligibility criteria to participate in the study.^21^ In order to ensure homogeneity of samples, patients who demonstrated Directional Preference (DP) for extension only were recruited. DP is described as the posture or movement that reduces or centralises radiating pain that originates from the spine. DP is important for the derangement group of patients with mechanical LBP.^21,22^ Patients with DP for flexion, positive history of red flags indicative of serious spinal pathology; any obvious spinal deformity or neurological disease; pregnancy; previous spinal surgery; and previous experience of MDT extension protocol, as well as, those with underlying systemic or visceral disease and specific condition such as dementia, cognitive dysfunction, visual impairment and previous history of epilepsy were excluded.

### Intervention

Each intervention comprised of warm up and the cool down phases. These phases involved a low intensity active stretching of the upper extremities, as well as the low back for about 5 min. The stretching exercises were carried out in a top-to-bottom sequence involving overhead triceps and arm-cross-chest stretch, forearm flexion and extension contraction and torso twist stretch performed in routine in a balanced standing position. Thereafter, the interventions were carried out by the participants in both groups.

The BE-VRG is an interactive video game which involves a less invasive three dimensional graphic environment on computer/television screen. The BE-VRG was developed and built into a Microsoft’s Kinect platform as virtual reality tasks. The choice and design of the tasks in the BE-VRG was based on the intention to achieve therapeutic activities that is comparable with the McKenzie ‘extension in standing’ protocol, as well as, the relevance of the tasks in rehabilitation of the patient. Participants in the BE-VRG group carried out the activities in an upright standing position with the feet slightly apart, while holding the waist with both hands. Then, they were asked to head virtual balls seen on the screen as though coming towards them, while their feet were stationary but the trunk and head moves to accomplish heading of the ball. The trajectory of the balls was set in such a way that it elicited the required therapeutic movements. The main therapeutic movement that was intended is trunk extension while standing. However, side-glide of the spine to the left and also to the right were accompanied movements during the tasks. This BE-VRG was designed to provide a progression of increasingly difficult challenges that can help keep the players (participants) engaged and motivated over extended period of time. During game play, visual and textual feedback on the patient’s performance and results were displayed on the screen.

Participants in the CBMT group received the McKenzie ‘extension in standing’ protocol. The protocol required that a participant stand upright with the feet slightly apart while placing his/her hands in the small of the back with the fingers pointing backwards.^23^ Then, the participant was asked to stretched the trunk backwards at the waist level as far as he/she could, using the hands as a fulcrum while keeping the knees straight. The protocol involves a course of specific lumbosacral repeated movements in extension that cause the symptoms to centralize, decrease or abolish.^23^ While the McKenzie extension protocol have different starting positions, however, in order to ensure comparability in orientation with the BE-VRG, the CBMT in this study was delimited to the standing position. The movement was repeated up to 10 times. Both interventions were carried out thrice weekly for 8weeks. Both groups received a set of back care education instructions comprising a 9-item instructional guide on standing, sitting, lifting and other activities of daily living for home.

### Outcomes and follow-up

All baseline data and measurements were recorded for each participant before and after randomisation. In addition, information such as age, gender, educational level, occupation, marital status, onset of back pain, recurrence, duration of complaint and previous intervention were recorded for each participant. Treatment health outcomes were assessed at the end of fourth and eighth week of the study. Oswestry Disability Index (ODI) was used to examine level of disability of the participants due from LBP.^24^ The tool covers 10 items including pain intensity, personal care, lifting, walking, sitting, standing, sleeping, sex life, social life and travelling. Each item scores from 0 (better) to 5 (worse). The ODI score was recorded for each participant and it was transferred in to a 0 to 100 scale. In order to estimate the health related quality of life of each participant to be used in economic evaluations of the interventions, the ODI score was mapped to SF-6D as described by Carreon and colleagues.^25^

The SF-6D instrument was used to provide estimation of utility using data from ODI, and this has enabled the researchers to perform a cost-utility analysis (CUA) (Brazier et al., 1998). CUA could be used to determine the cost in terms of utilities, and it combines the quantity and quality life. After obtaining the SF-6D values of each participant, the quality adjusted life year (QALY) of each participants was calculated. QALY was calculated by multiplying the SF-6D values and the duration of time (years). For the purpose of this study, the average of QALYs at 4 weeks and 8 weeks period of each participant was considered.

### Resource use and costs

Healthcare resource use and costs were assessed from the health system perspective. The direct healthcare resources for implementing the BE-VRG and CBMT were: back treatment, consultation, development of Microsoft connect device and flat screen monitor, development of BE-VRG, refreshment and consumables. Costs were measured in Nigerian Naira and US$ using the 2021 currency conversion rate (US$1 = ₦367). Patient participants were the sources of all the healthcare resource uses included for this study. These resources were documented from McKenzie therapy protocols. Personal costs associated with CBMT was not included in this analysis. As the patients were those attending outpatient physiotherapy departments, cost of medications were not included in this study. Moreover, in the context of this study, most of the participants were able to access healthcare through out-of-pocket means, in addition to undisclosed self-medication practices that is often encouraged by over the counter access to more than the regulated medications. Productivity lost by the patient was not quantified. Costs are reported to the nearest Nigerian Naira and US$.

### Statistical and cost-effectiveness analysis

Descriptive statistics of the mean or standard deviation and inferential data analysis were performed using the Statistical Package for the Social Sciences for Windows (version 23). Mann–Whitney U test and Friedman tests were used to compare the mean effects between the treatment regimen across the fourth- and eighth-week period and the changes of the effects of the interventions from baseline at the fourth week and eighth week for the categorical variables, respectively. A significance level of *p* = .05 was adopted for those comparisons. Incremental cost-effectiveness ratio (ICER) was used to assess the cost-effectiveness of TBMT compared to CBMT using the formula below.^26^

$$\begin{array}{l} \text{ICUR}\;\;\;\;=\Delta \text{Cost}/\Delta \text{Effectiveness}\\ =\left({\text{Cost}}_{\text{BE}-\text{VRG}}-{\text{Cost}}_{\text{CBMT}}\right)/\left({\text{QALY}}_{\text{BEVRG}}-{\text{QALY}}_{\text{CBMT}}\right) \end{array}$$

The incremental cost-effectiveness ratio is the differential costs and outcomes between new intervention (BE-VRG) and the control (CBMT).^27^ The numerator in the cost-effectiveness ratio is the monetary cost of the TBMT intervention minus the monetary cost of CBMT. The annual costs of the projects were calculated by converting the 8 weeks costs, the time period used for implementation. The denominator is the QALY gained by BE-VRG minus the QALY gained by CBMT. Bootstrapping was used for pair wise comparison for the mean costs and effects between the BE-VRG and CBMT groups. Confidence intervals for the mean differences in effects were obtained by bootstrapping (1000 replications). The bootstrapped costs and effects pairs were also graphically represented on a cost effectiveness plane.^28^

## Results

**Figure 1.**
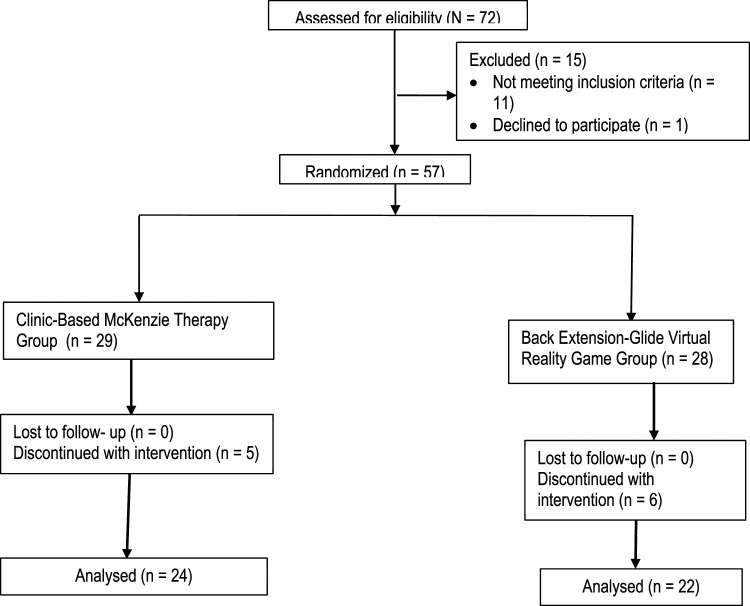
Consort diagram of the flow of patients through the study.

Forty-six patients (BE-VRG, *n* = 22; CBMT, *n* = 24) with the mean (±SD) age of 47.62 ± (11.5) years for BE-VRG and 48.8± (10.2) years for CBMT intervention completed in this study. A total of five and six participants in the CBMT and BE-VRG, respectively, have discontinued with the interventions (Figure 1).

### Effectiveness

Table 1 reports the functional disability of participants as measured by the ODI. The mean (SD) ODI scores at baseline (BE-VRG, 14.23 (9.41); CBMT, 21.12 (10.68)), week 4 (BE-VRG, 6.73 (6.05); CBMT, 12.67 (5.61) and week 8 (BE-VRG, 3.54 (3.53); CBMT, 6.75 (5.06)) are presented in Table 1. Functional disability of the participants between the BE-VRG and CBMT was significantly different (*p* < .05) at baseline, week 4 and week 8. Over the 8 weeks period, there was an indication that functional disability of participants has improved in the two arms.

**Table 1.** Scores of Oswestry disability index.

ODI score	BE-VRG (*n* = 22) Mean (SD)	CBMT (*n* = 24) Mean (SD)	Mean difference (95% CI)	*p* value
Baseline	14.23 (9.41)	21.12 (10.68)	6.89 (0.89–12.9)	.025
At week 4	6.73 (6.05)	12.67 (5.61)	5.94 (2.5–9.4)	.001
At week 8	3.54 (3.53)	6.75 (5.06)	3.2 (0.59–5.82)	.017

ODI: Oswestry Disability Index; BE-VRG: Back Extension- Virtual Reality Game; CBMT: Clinic-based McKenzie therapy; SD: Standard deviation; CI: Confidence interval.

### Resource use and cost

We estimated the mean direct cost of the BE-VRG and CBMT over the 8 weeks period of time (Table 2). A total of 24 treatment sessions (₦500 (US$1.39) per sessions) and 10 additional treatments to those participants coming for the first time have been provided. The costs estimate for SMS messages & reminder calls (3 times a week) were ₦50 (US$0.14) per unit. The costs estimate of clinic visit (3 visits per week) for CBMT was *N*1,000 (US$2.78) per visit, and transportation and refreshments for each clinic visit estimate was ₦500 (US$1.39) per visit. The total costs estimate for the development VRG and purchase of Microsoft connect device and flat screen monitor were US$1018 and US$971.00, respectively. Moreover, the common costs to both groups were costs of physiotherapy consultation (before randomisation into group), and were estimated ₦1000.00 (US$2.78). Patients allocated to the BE-VRG group had slightly lower costs than CBMT.

**Table 2.** Total cost per patient associated with implementation of back extension-virtual reality game and clinic-based McKenzie therapy over 8-week period.

Services	BE-VRG (*n* = 22)		CBMT (*n* = 24)		Cost per patient (₦)	
	Quantity	Cost (₦)	Quantity	Cost (₦)	BE-VRG (*n* = 22)	CBMT (*n* = 24)
Treatment sessions	34 sessions	₦17000			US$2.1	
Consultation fee	1 case	₦550			US$2.8	US$2.8
SMS messages & reminder calls (3 times a week)	—	—	—	—	—	US$3.4
Development of BE-VRG	—	US$1018			US$46.27	—
Clinic visit			24 visit			US$66.7
Development of Microsoft connect and flat screen monitor	—	US$971	—	—	US$44.1	—
Transportation & refreshment	2 visits	US$2.8	24 visit		US$5.6	US$33.4
Total					US$100.87	US$106.3

BE-VRG: Back Extension- Virtual Reality Game; CBMT: Clinic-based McKenzie therapy; US$: United States Dollar; ₦: Nigerian Currency.

### Incremental cost-effectiveness ratio

Table 3 shows the cost-effectiveness analysis. The total costs of the BE-VRG was US$5.6 per patient lower than the CBMT. This difference was primarily due to the reduced or no clinic visit, transportation and refreshment costs such as food and drinks. Parallel to this, the mean quality adjusted life years (QALY) values for the CBMT and BE-VRG were 0.084 [95% CI 0.083–0.086] and 0.087 [95% CI 0.086–0.088]. There was 0.003 [95% CI 0.001–0.004] QALY gain by patient assigned in the BE-VRG compared to CBMT. Though these QALY differences are small, the adjusted analysis showed a statistically significant difference using independent t-test favouring the BE-VRG intervention (*p* = .003). The ICER values indicate that the healthcare costs and QALY favoured the BE-VRG. In other words, BE-VRG had lower costs and better patient outcomes compared to CBMT and the calculation of the ICER is inappropriate as it can be misleading.

**Table 3.** Incremental cost-effectiveness analysis.

	Cost (US$)	Incremental cost (US$)	Effect mean (95% CI) QALY	Incremental effect, mean (95%CI), QALY gain	Incremental cost-effectiveness ratio (US$)/QALY)
Clinic-based McKenzie therapy	106.3	—	0.084 (0.083–0.086)	—	—
Back extension- virtual reality game	100.87	−5.6	0.087 (0.086–0.088)	0.003 (0.001–0.004)	Dominant

CI: Confidence interval; QALY: Quality Adjusted Life Year; US$: United States Dollar.

**Figure 2.**
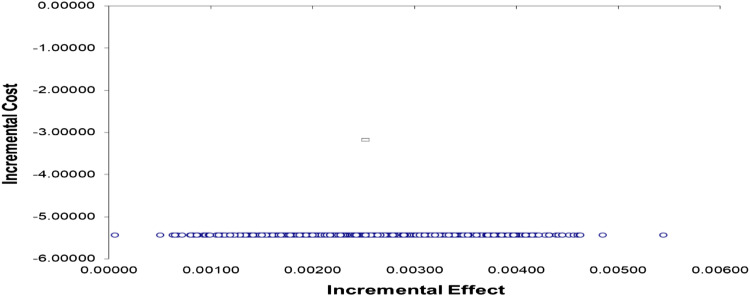
Incremental cost-effectiveness plane.

Figure 2 shows the incremental cost effectiveness plane for a plot of 1000 bootstrap incremental costs and effects resample means. All of the resample means are located in the southeast quadrant of the plane, with negative costs and positive effects. The plane demonstrates that BE-VRG is always considered cost-effective.

## Discussion

To our knowledge, this is the first economic evaluation of BE-VRG interventions for chronic LBP. Compared to the CBMT, the BE-VRG lead to the reduced healthcare costs and increase in QALYs for patients with chronic LBP. We estimated the incremental cost effectiveness ratio to be dominant. However, it is important to note that adherence of patients to the treatment for better outcomes and or much shorter contact times are the factors that might have contributed to the cost-effectiveness of BE-VRG compared with CBMT.

This study showed that there was a significant difference (*p* < .05) in the scores of ODI between BE-VRG and CBMT at baseline, weeks 4 and weeks 8. A meta-analysis of randomised controlled trials have also indicated that the McKenzie method and video games are more effective than passive therapy showed a statistically significant difference in pain and disability.^29,30^ Further, a moderate evidence for the reduction of pain and functional impairment was found in patients with acute pain when video-based virtual reality system was used as an adjunct therapy.^31^ However, these studies have emphasised that there was limited evidence within the included studies for adherence and effectiveness specifically in the long-term trials.

In the current study, the cost-effectiveness analysis is largely supportive of the BE-VRG intervention given the current evidence and compared to CBMT alternative. This is because of the BE-VRG intervention was both more effective at producing health benefits than the CBMT and were associated with net cost savings. However, there are a number of limitations to this study. Firstly, the adoption of health system perspective in this study have excluded patients’ time and personal costs and loss of production due to treatment and poor health. The authors believe that the cost-effectiveness of the intervention could have been different if societal perspective was used. Secondly, the imbalance between groups at baseline in function as measured with ODI could be by chance due to the characteristics of the patients.^32^ Thirdly, the cost of implementation for BE-VRG and CBMT were not relied upon information collected from each participants; this may have an impact on its level of reliability due to cost under or over estimation. Lastly, due to variation in clinical practices, and absolute and relative differences in price weights among different countries, these may limit the generalisability of the findings of the current study. Furthermore, it is possible that the additional motions of the trunk in gliding (right and left) to the extension movement would give added effects and in turn influence outcomes in the BE-VRG group. This is because the gliding movements are also important therapeutically. McKenzie describes side-gliding as the simultaneous combination of lumbar rotation and side-bending.^33^ This movement is thought to produce relief of pain among patients with LBP.^34^ Lastly, the McKenzie extension in standing’ is one of the most common therapies utilised by physical therapists in active rehabilitation of patients with LBP in the clinical settings, as well as in self-care prescriptions for home programmes. Also, most therapeutic games are typically carried out in the ‘standing’ fundamental position. Thus, comparing the outcome of the BE-VRG utilized in this study should be delimited to findings of studies that employed the McKenzie ‘extension is standing’ only. Overall, there is a considerable potential for more research to be considered in the future in the longer term and from societal and patient perspective as healthcare is largely out of pocket in LMIC, and LBP is hugely associated with productivity loss.

## Conclusion

The findings of this study suggest that VRG was cost saving in the management of chronic non-specific LBP compared to CBMT from healthcare perspective. This evidence could guide policy makers, payers and clinicians to consider BE-VRG for people with chronic non-specific LBP as it seems to improve health related quality of life at lower costs. The findings can also provide support for the global health initiative of improving access to physiotherapy interventions for patients with LBP in low and middle-income countries.
